# 1328. Risk Factors for Severe Influenza Outcomes Among Infants Born Between 2011 and 2019

**DOI:** 10.1093/ofid/ofab466.1520

**Published:** 2021-12-04

**Authors:** Sarah Chamseddine, Haniah A Zaheer, John V Williams, Judith M Martin, Anne-Marie Rick, Hui Liu

**Affiliations:** 1 UPMC Children’s Hospital of Pittsburgh, Pittsburgh, Pennsylvania; 2 University of Pittsburgh School of Medicine, Pittsburgh, Pennsylvania; 3 University of Pittsburgh, Pittsburgh, Pennsylvania

## Abstract

**Background:**

Influenza in infancy can cause significant morbidity and mortality. This study aimed to characterize influenza outcomes in infants < ορ = 12 months and identify risk factors for severe infection.

**Methods:**

A retrospective cohort of infants ≤ 12 months born between 2011-2019 who received longitudinal ambulatory and inpatient care within a multi-facility hospital system and had laboratory-confirmed influenza were included. Perinatal, medical and illness characteristics were described. Risk factors for severe influenza (hospitalization, intensive-care unit (ICU) admission, secondary bacterial infections) were analyzed using Chi-square analysis and multivariate logistic regression.

**Results:**

Among 421 infants with influenza, 134 (32%) were < 6 months (m), 28 (6.5%) were born prematurely (< 35 weeks gestational age), and 41(10%) had chronic medical conditions (CMC). 62 (15%) required hospital admission, 13 (21%) of which required ICU care. No deaths were reported. Secondary bacterial infections were diagnosed in 101 (24%) including acute otitis media (84%), pneumonia (15%) and sinusitis (3%). Prematurity (OR 3.6, 95%CI:1.5-8.3), age < 6m (OR 3.4, 95%CI:1.9-5.9), and CMC (OR 7.6, 95%CI 3.8-15.3) were significantly associated with hospitalization. Prematurity, age < 6m, and CMC were also associated with ICU admission. Infants > 6m (OR 2, 95%CI:1.2-3.5) were more likely to be diagnosed with a secondary bacterial infection than younger infants. Among infants > 6m, complete influenza vaccination (2 doses) was associated with lower rates of antibiotic use (OR 0.5, 95% CI:0.3-0.9) compared to partial or no vaccination, but did not significantly affect hospitalization, ICU admission, or frequency of secondary bacterial infections. Adjusting for prematurity, age < 6m remained associated with hospitalization (aOR 4, 95%CI: 2.1-7.3) as did presence of CMC (aOR 7.3, 95%CI 3.3- 15.7). For ICU admission, age < 6m (aOR 6.3, 95%CI:1.6-24.1) and CMC (aOR 19.7,95%CI:4.9-79.5) were also independent risk factors.

**Conclusion:**

Younger age and chronic medical conditions were independent risk factors for severe influenza infection. Complete influenza vaccination in eligible age groups was associated with decreased antibiotic use.

**Disclosures:**

**John V. Williams, MD**, **GlaxoSmithKline** (Advisor or Review Panel member, Independent Data Monitoring Committee)**Quidel** (Advisor or Review Panel member, Scientific Advisory Board) **Judith M. Martin, MD**, **Merck Sharp and Dohme** (Consultant)

